# Oxidative Stability of Sunflower Oil: Effect of Blending with an Oil Extracted from Myrtle Liqueur By-Product

**DOI:** 10.3390/antiox14030300

**Published:** 2025-02-28

**Authors:** Daniele Sanna, Angela Fadda

**Affiliations:** 1 Institute of Biomolecular Chemistry, National Research Council, Traversa La Crucca, 3, 07100 Sassari, Italy; 2 Institute of the Sciences of the Food Productions, National Research Council, Traversa La Crucca, 3, 07100 Sassari, Italy

**Keywords:** sunflower oil, oil blends, myrtle by-products, oxidative stability, forced aging, Electron Paramagnetic Resonance (EPR) spectroscopy

## Abstract

Myrtle oil extracted from the spent berries of myrtle liqueur production, using 2-methyltetrahydrofuran, was used to increase the oxidative stability of sunflower oil (SFO). Three blending ratios (5%, 10%, and 15% *w*/*w*) and the SFO without any addition were subjected to forced aging conditions at 70 °C for 21 days. The changes in peroxide value (PV), p-anisidine value (AV), total oxidation value (totox), and conjugated dienes and trienes were evaluated during forced aging. The oxidative stability of the blends was also assessed by the spin trapping method coupled with Electron Paramagnetic Resonance spectroscopy. Myrtle oil at 5% provided the best results, increasing the oxidative stability of SFO by reducing the PV and slowing the onset of secondary oxidation products, as measured by the AV and conjugated trienes. The 15% blend, despite its high levels of PV, AV, conjugated dienes, and trienes during storage, protects SFO from oxidation. The blends of SFO with unconventional oils, like myrtle oil, could represent a sustainable approach to increase its oxidative stability during storage.

## 1. Introduction

Sunflower oil (SFO) is one of the most widely used edible oils in the world. Global SFO consumption is forecast to exceed 20.27 million tons in 2024 and has been increasing over the past decade [1]. Its commercial success is due to its nutritional properties, with high levels of oleic and linoleic acids, which are known to decrease cholesterol levels and prevent heart disease. However, the disadvantage of replacing saturated oils with unsaturated ones, which are perceived as healthier, is an increased susceptibility to oxidation. Internal factors, such as the fatty acid profile and the presence of antioxidants, together with external factors, such as temperature and oxygen availability, affect the oxidation of oils [2].

To slow down the oxidation process, the edible oils endowed with low levels of antioxidants, including SFO, are fortified with vitamin E or synthetic antioxidants, such as butylated hydroxyanisole (BHA) or butylated hydroxytoluene (BHT) [3]. Nevertheless, the potential risk of safety associated with synthetic antioxidants has led to the development of methods based on the use of natural antioxidants to delay oxidation. There are several studies in the literature on the effect of phenolic compounds as alternatives to synthetic antioxidants to control oil oxidation during storage or cooking [4,5,6,7,8,9,10,11].

Another method to improve oils’ oxidative stability is oil blending. The blending of two or more oils has the purpose of enhancing the oils’ quality by modifying the fatty acid profile and increasing the antioxidant concentration, thus improving the functional properties and the oxidative stability [3].

The number and the characteristics of the oils employed in the various blends can vary considerably, from two to four or even more, encompassing a wide range of oil species. The choice of the oils and the ratio of each oil (formula) depend on the desired outcome or the food application. Blending influences the fatty acid profile, in particular, the ratio of saturated, mono- and polyunsaturated fatty acids and the ratio of essential fatty acids (ω3/ω6). For example, blending SFO with 20% cold-pressed flaxseed oil resulted in an oil with an optimal ω3/ω6 ratio and an increased level of bioactive compounds, such as tocopherols and carotenoids [12]. More recently, the blending of peanut oil with 30% walnut oil rich in ω3 fatty acid produced a blend with an optimal ω3/ω6 ratio and an improved oxidative stability [13].

Another important effect of the blending process is the improvement of oxidative stability. Blending lineseed oil with corn, sesame, and bitter almond oils enhanced the oxidative stability of lineseed oil, as determined by the Rancimat method at 353, 373, and 393 K [14]. Similarly, the blend of SFO with pomegranate seed oil (85:15) showed an increase in oxidative stability during the accelerated storage test at 60 °C for 20 days, compared to SFO alone [15].

Blending is a traditional method that has recently regained interest due to the presence of unconventional oils on the market. Blending SFO with the oil extracted from myrtle seeds, as proposed in this study, follows this trend.

Myrtle is an evergreen shrub used in Europe to extract essential oils and to produce myrtle liqueur by the hydroalcoholic infusion of its berries. Each year, the production of myrtle liqueur generates a large amount of by-products, mainly pulp and seeds, which are currently unexploited, being used as combustible material or considered waste. With the aim of valorizing this precious material, our previous work has focused on the sustainable extraction, with biobased, non-toxic, and biodegradable solvents, of a myrtle seed oil (MSO), studying its fatty acid profile, the antioxidant properties, and the oxidative stability [16]. The composition of the MSO extracted with 2-methyltetrahydrofuran has already been reported by Fadda et al. [16].

In this paper, we aimed to evaluate the use of MSO extracted with 2-methyltetrahydrofuran as a sustainable method to improve the oxidative stability of SFO under forced aging conditions (70 °C for 21 days). The oxidative stability during the storage of SFO and the blends was also studied using the spin trapping technique coupled with Electron Paramagnetic Resonance (EPR) spectroscopy, as previously reported [16,17].

## 2. Materials and Methods

### 2.1. Chemicals

All reagents and solvents were of analytical grade unless otherwise specified and used without further purification. Additionally, 2-methyltetrahydrofuran (2-MeTHF), N-tert-butyl-α-phenylnitrone (PBN), p-anisidine, chloroform, methanol, isooctane, iron(III) chloride, iron(II) sulfate heptahydrate, and ammonium thiocyanate were purchased from Sigma-Aldrich (Milan, Italy). Ultrapure water was prepared using a Milli-Q system (Millipore Corporation, Billerica, MA, USA).

### 2.2. Vegetable Oil Samples

SFO was purchased from the local retailer. According to the manufacturer’s label, the oil contains 10.9% *w*/*w* saturated fatty acids (mainly palmitic and stearic acid), 30.4% *w*/*w* monounsaturated fatty acids (mainly oleic acid), 58.7% *w*/*w* polyunsaturated fatty acids (mainly linoleic acid), and 450 mg/L vitamin E.

MSO was obtained from the seeds of myrtle berries, by-products of myrtle liqueur production, which were kindly provided by a local liqueur processing industry. Upon arrival at the laboratory, spent berries were inspected to remove spoiled biomasses and then air-dried. The pulp and seeds were separated with a laboratory seed air cleaning machine and seeds were stored until oil extraction.

As previously reported by Fadda et al. [16], cold extraction was carried out with 2-MeTHF. This solvent was chosen because it is able to extract both phenolic compounds and oils due to its medium polarity and because it is environmentally sustainable. For the extraction, 30 g of milled seeds was mixed with 300 mL of 2-MeTHF and left under continuous stirring for 24 h at room temperature. The extracts obtained were filtered to remove solid residues, and the organic solvent was evaporated under vacuum. The oil recovered was weighed and oil yield was calculated as the percentage (*w*/*w*) of oil over seed weight. Three separate extractions were performed. The oils obtained were stored at −20 °C until blending with SFO. The chemical composition, the concentration of phenolic compounds, the antioxidant activity, and the oxidative stability evaluated with the EPR spin trapping method have been reported in our previous work [16].

### 2.3. Preparation of Oil Blends and Storage Conditions

Sunflower and myrtle oils were mixed in three proportions by weight: 5, 10, and 15%, and then 2 mL of each blend was put into vials with an inner diameter of 18.15 mm. The choice of the blending ratio was based on a previous paper available in the literature dealing with unconventional seed oils [15].

For each blend, the vials were randomly arranged into six groups of three vials each (each vial was a replicate). The first group was used as a control and not subjected to heating conditions, while the others were heated at 70 °C in an oven for 2, 4, 7, 14, and 21 days to simulate the storage conditions at room temperature for longer time intervals.

At the end of each storage time, oils were analyzed for peroxide value, p-anisidine value, conjugated dienes and trienes, and oxidative stability through Electron Paramagnetic Resonance (EPR) spin trapping analysis.

### 2.4. Determination of Peroxide Value (PV)

Peroxides were determined according to Hornero-Méndez et al. [18], with some modifications. In total, 10 mg of SFO, myrtle oil, and their blends was dissolved in 9.8 mL of chloroform-methanol 7:3 (*v*/*v*) then 50 µL of ammonium thiocyanate (394 mM) and 50 µL of FeSO_4_ × 7H_2_O (18 mM) were mixed. The absorbance was measured at 507 nm with a Perkin-Elmer Lambda 35 spectrophotometer (PerkinElmer, Shelton, CT, USA) after 15 min of storage in the dark. Peroxide value was expressed as meq O_2_/kg of oil based on a calibration curve built using FeCl_3_ as an Fe(III) source (Fe(III): 1.2 × 10^−5^−9.3 × 10^−5^ M; R^2^ = 0.99). Data are presented as mean, calculated considering three replicate values, ± standard deviation.

### 2.5. p-Anisidine Value (AV)

p-Anisidine values were determined according to the AOCS official method Cd 18-90 [19]. The p-Ansidine was purified before use by dissolving 40 g of it in 1 L of water at 75 °C. In addition, 2 g of sodium sulphite and 20 g of active carbon were added while stirring for 5 min and were then filtered. The filtered solution was cooled at 0 °C and the crystallized p-anisidine was filtered off, dried in a vacuum desiccator, and stored in the dark at a low temperature. For the determination of the p-anisidine value, ca. 0.5 g of SFO and its blends with MSO extracted with 2-MeTHF were exactly weighted and dissolved in 5 mL of isooctane. The absorbance of these solutions was read at 350 nm (A_b_). Additionally, 2 mL of oil solutions in isooctane or 2 mL of isooctane was mixed with 0.4 mL of a p-anisidine solution in glacial acetic acid (2.5 g/L). After 10 min, the absorbance of oil solutions containing p-anisidine (A_s_) was read at 350 nm using the solution of p-anisidine diluted with isooctane as a blank.

The p-anisidine values were obtained with the following formula:

AV = [5 × (1.2A_s_ − A_b_)]/m, where m is the mass in grams of the weighted oils.

Data are presented as mean, calculated considering three replicate values, ± standard deviation.

### 2.6. Conjugated Dienes and Trienes

The spectroscopic indexes K232 and K268 were determined according to the method proposed by the International Olive Oil Council [20]. Samples were prepared using solutions of the oils in isooctane. The specific absorbance values at 232 and 268 nm were recorded with a Perkin-Elmer Lambda 35 spectrophotometer against a blank of pure isooctane in 1 cm optical path-length UV cuvettes. Data are presented as mean, calculated considering three replicate values, ± standard deviation.

### 2.7. EPR Spin Trapping Analysis of Oil Blends: EPR Settings and Spectra Acquisition

The oxidative stability of oil blends was determined, as previously reported, by the spin trapping method coupled with EPR spectroscopy [16]. The PBN spin trap was dissolved in ethanol to obtain a final concentration of 2.5 M. Additionally, 5 µL of this solution was dried under a nitrogen flow then 100 µL of oil was mixed with the solid PBN, transferred into an EPR quartz tube, and inserted into the resonant cavity heated at 90 °C. Spectra acquisition lasted 3 h, with spectra taken every 5 min. The intensity of the PBN adduct was estimated from the double integration of the spectra and was plotted against time. At least two replicates were analyzed for each oil sample.

A Bruker EMX spectrometer (Bruker, Billerica, MA, USA) operating at the X-band (9.4 GHz) equipped with an HP 53150A frequency counter and a variable temperature unit ER 4111 VT was used to measure the radical adducts during the oils’ thermal treatment. Spectra were acquired with Bruker WinEPR Acquisition Version 4.33. The EPR instrument was set under the following conditions: modulation frequency 100 kHz, modulation amplitude 0.106 mT, receiver gain 5.02 × 10^4^, microwave power 20 mW (which is with the ER 4119HS cavity, below the saturation limit), resolution 1024 points, sweep time 167.772 s, and a time constant and conversion time of 163.84 ms. The selected values of time constant, sweep time, resolution, and sweep width allowed us to resolve the narrowest line corresponding to 0.049 mT.

### 2.8. Statistical Analysis

Statistical analysis was performed with GraphPad Prism 8 for Windows software (GraphPad Software Inc., La Lolla, CA, USA). Analysis of variance (ANOVA) was performed according to a single factor, randomized block design. Mean comparisons of the effects of the blending treatment within each forced aging time and the influence of forced aging time within each oil were calculated, and were applicable, by Tukey’s test (*p* ≤ 0.05).

## 3. Results and Discussion

### 3.1. Chemical Properties of Myrtle Seed Oil (MSO)

The quality and the chemical properties of the oil extracted with 2-MeTHF from myrtle seeds were reported in our previous paper [16]. Additionally, 2-MeTHF is a solvent of medium polarity capable of simultaneously extracting lipids and phenolic compounds. Indeed, the MSO extracted with 2-MeTHF had a high concentration of phenolic compounds and a high radical scavenging activity, probably due to the high concentration of hydrolysable tannins and flavonoids. The PV of MSO was 34.77 ± 1.85 meq O_2_/kg. This value is relatively high because MSO is not refined.

### 3.2. Peroxide Value (PV)

**Figure 1:**
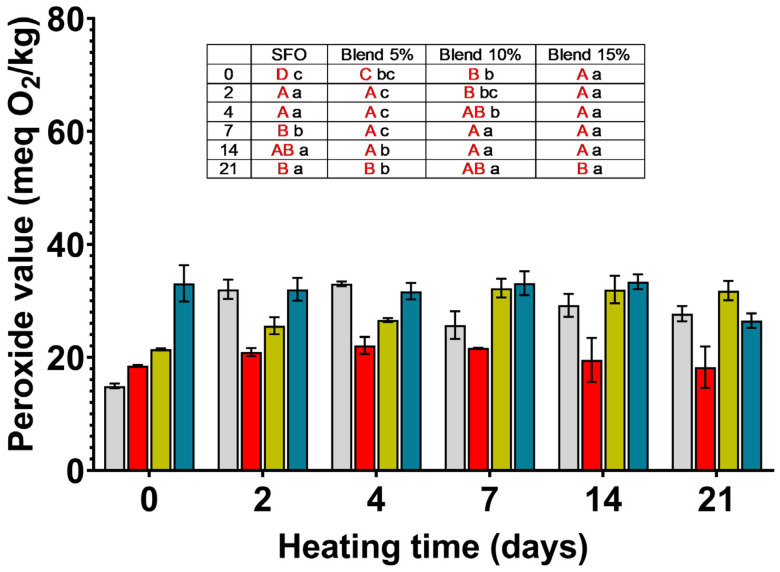
Peroxide values (meq. O_2_/kg) measured for SFO and its blends (5, 10, and 15%) with MSO extracted with 2-MeTHF during storage at 70 °C for 21 days. 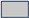 SFO; 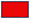 blend 5%; 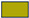 blend 10%; 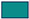 blend 15%. In the inset table: capital letters in red deal with the comparison between times inside the same blend; lowercase letters deal with the comparison between blends inside the same storage time. Mean differences were calculated according to Tukey’s test (*p* ≤ 0.05).

The changes in PV during storage are shown in Figure 1. Peroxide value (PV) measures the primary oxidation products and is a marker of oil quality. Oil’s internal and external factors, like the degree of unsaturation, the oxygen availability, the storage temperature, and the method of oil extraction, affect the PV [16]. The recommended maximum peroxide value (PV) for an alimentary oil is established by the Codex Standard for Named Vegetable Oils [21], amended for the last time in 2024; this value is up to 10 meq O_2_/kg in refined oils and up to 15 meq O_2_/kg in cold-pressed and virgin oils. The control oil (SFO not exposed to accelerated aging conditions) had a PV of 14.92 ± 0.42 meq O_2_/kg below the limit set for edible oil, whereas MSO extracted with 2-MeTHF had a PV about 2.3 times higher than SFO.

In the blends not exposed to aging conditions, the PV was significantly higher than in the SFO control oil. This is due to the effect of MSO, which, as mentioned above, has higher peroxide levels.

In SFO, storage conditions (70 °C) caused an increase in PV, which reached a maximum value on the fourth day of accelerated aging and then decreased but never returned to the initial levels. During storage, the PV increased for all samples except for the blend with 15% myrtle oil, which remained unchanged and decreased only after 21 days of storage.

The antioxidants present in MSO protected the blends from oxidation during accelerated storage. Indeed, when comparing the increase in peroxides in pure SFO and blends, the greater increase is observed with pure SFO, suggesting that in blends, the antioxidants, originally present in the added MSO extracted with 2-MeTHF, protect them against peroxidation. The protective effect was evident for the 5% blend from the second day of storage and continued throughout the entire storage period. The protection provided by MSO was not concentration-dependent, as observed for the 10% and 15% blends. As noted above, the 15% MSO blend had PVs significantly higher than the control throughout the storage period. However, the graph (Figure S1) describing the percentage increase in PV during storage, relative to time 0, shows that there is no increase in the 15% blend, highlighting the protective effect of the antioxidants. The 10% blend showed PVs lower than the control up to the fourth day of storage, then they increased, clearly showing two well-differentiated stages.

A high PV is not a marker for off-flavors. As underlined by Talbot [22], peroxides PVs are the precursors of the changes that occur during oil oxidation. Even in highly oxidized oil, PV can be low because they have been decomposed into aldehydes and ketones. This can be observed in our experiments as well. After 21 days of storage at 70 °C in SFO and in all the blends, the PVs were lower than at the beginning of the experiment.

### 3.3. Dienes and Trienes

**Figure 2:**
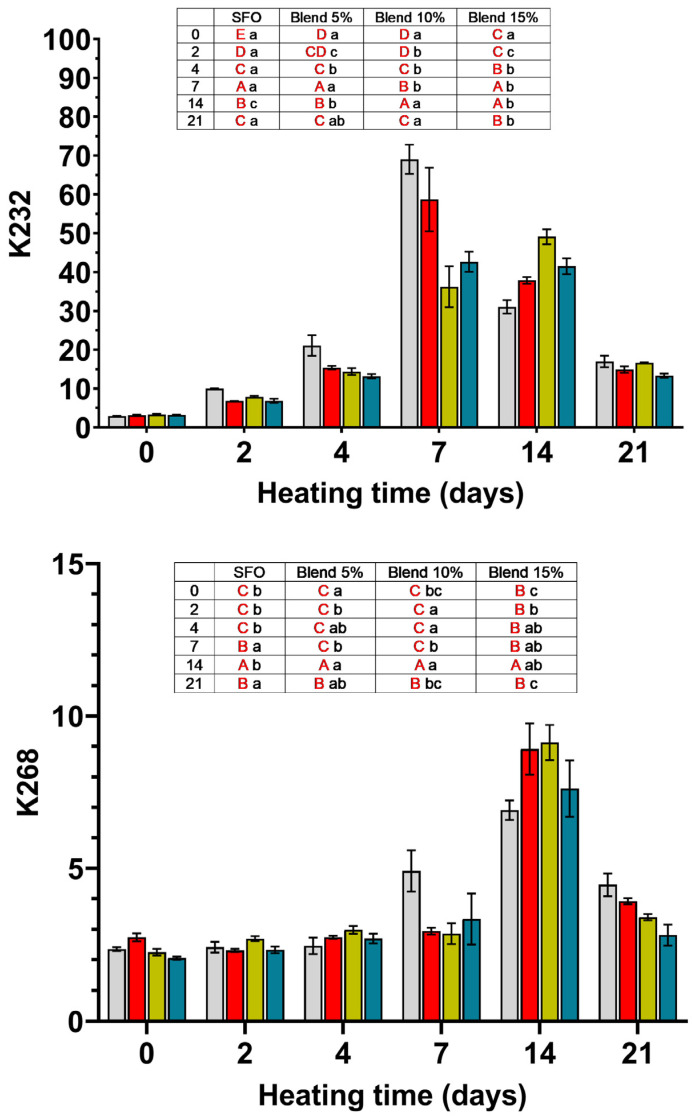
K232 and K268 values measured for SFO and its blends (5, 10, and 15%) with MSO extracted with 2-MeTHF during storage at 70 °C for 21 days. 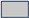 SFO; 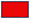 blend 5%; 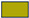 blend 10%; 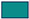 blend 15%. In the inset table: capital letters in red deal with the comparison between times inside the same blend; lowercase letters deal with the comparison between blends inside the same storage time. Mean differences were calculated according to Tukey’s test (*p* ≤ 0.05).

The K232 and K268 values are reported in Figure 2. These values are an index of the formation of conjugated double (dienes) and triple bonds (trienes) during oxidation, with strong absorption maximums at 232 nm and at 268 nm, respectively.

According to the literature [23,24], K232 values show a good correlation with hydroperoxydienes, indicating that these are the main compounds absorbing at 232 nm. In a high linoleic SFO with composition (palmitic acid 6.7%; stearic acid 3.6%; oleic acid 33.0%; linoleic acid 55.2%; others 1.5%) similar to that of the SFO examined in the present work, the hydroperoxides are mainly hydroperoxydienes derived from linoleic acid [23]. The SFO used in this paper had a composition similar to that reported in the cited paper but such a correlation was not present, not even in the first days of storage, indicating that the dienes determined in this paper also arise from conjugated dienes non-hydroperoxides [25]. This difference is probably due to the presence of vitamin E in our SFO, while the oil used in the cited paper was deprived of tocopherols, and the different temperatures to which the two oils were subjected: 70 °C in our case and 40 °C in the cited paper. This can deeply affect the evolution of oil in terms of oxidation products because the solubility of oxygen considerably changes. In conditions of limited oxygen solubility, the C-centered radical species derived from fatty acids, once formed, instead of reacting with oxygen forming hydroperoxydienes, form polymeric species that retain their conjugated double bonds. This could explain why there is no relationship between the PV and K232 values.

Conjugated dienes measured in SFO and blends showed a maximum at 7 days of storage. Up to the fourth day of accelerated aging, no differences were observed between SFO and any of the blends while, at day seven, SFO had a conjugated diene value significantly higher than the blends. The trienes show a similar trend but with a delay of the highest values shifted to the 14th day of storage. Such a sudden increase may be the result of the decomposition of the peroxides, which takes place from the 7th day of storage at 70 °C. The dienes and trienes increased significantly from days 7 and 14, respectively.

### 3.4. p-Anisidine Value (AV)

**Figure 3:**
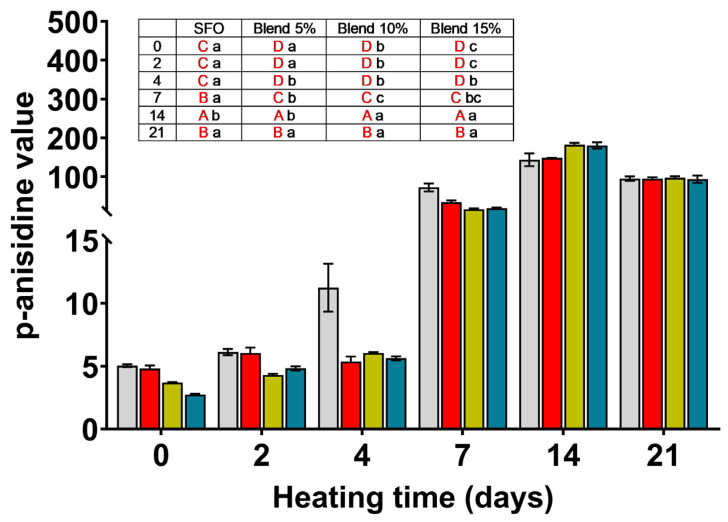
The p-Anisidine values measured for SFO and its blends (5, 10, and 15%) with MSO extracted with 2-MeTHF during storage at 70 °C for 21 days. 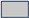 SFO; 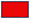 blend 5%; 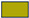 blend 10%; 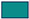 blend 15%. In the inset table: capital letters in red deal with the comparison between times inside the same blend; lowercase letters deal with the comparison between blends inside the same storage time. Mean differences were calculated according to Tukey’s test (*p* ≤ 0.05).

The p-anisidine values are reported in Figure 3. Unlike the PV, the AV is a marker of the final stages of oil oxidation and measures the aldehyde and ketone breakdown products of peroxides, which are responsible for oil’s off-flavors. Therefore, the lower the AV, the better the quality of the oils analyzed.

According to Talbot, oils with a AV less than 10 are acceptable [22]. The SFO kept the AV below the limit up to the second day of storage then AV increased progressively until the end of storage. The blends showed the AV below the acceptable limits up to the fourth day of accelerated aging, suggesting a delay in the oxidation process. No or little differences were observed among the blends with different percentages of MSO within each storage period. Considering the changes in peroxides and p-anisidine values during storage, it can be hypothesized that peroxides, which accumulate during the first stages of storage at 70 °C, begin to break down before 7 days of storage, causing an increase in p-anisidine values.

### 3.5. Totox Value (TV)

To give a complete picture of oil oxidation, a value mixing the information provided by the PV and AV was developed. The totox value is an arithmetical formula that combines twice the PV and AV [22].

**Figure 4:**
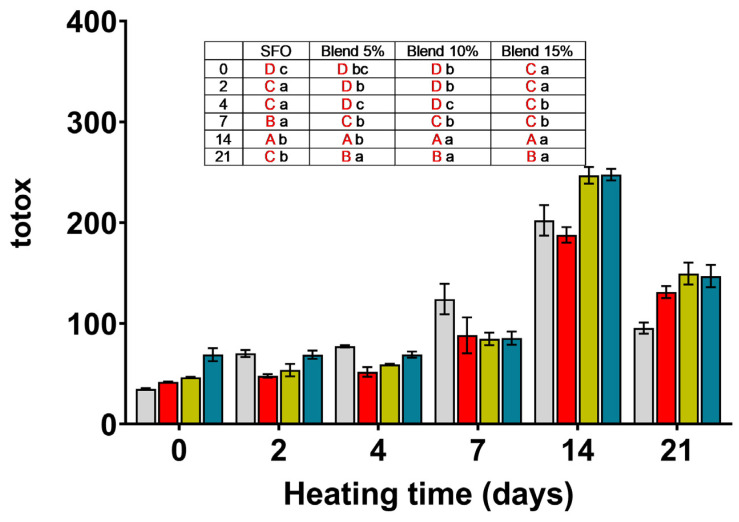
Totox values calculated for SFO and its blends (5, 10 and 15%) with MSO extracted with 2-MeTHF during storage at 70 °C for 21 days. 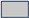 SFO; 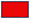 blend 5%; 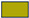 blend 10%; 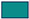 blend 15%. In the inset table: capital letters in red deal with the comparison between times inside the same blend; lowercase letters deal with the comparison between blends inside the same storage time. Mean differences were calculated according to Tukey’s test (*p* ≤ 0.05).

In SFO and in the blends, the totox value increased progressively with storage, reaching in SFO the highest value at 14 days. (see Figure 4). According to totox values, the blends were all effective in controlling oil oxidation after 2, 4, or 7 days of storage. Little or no difference between the blend percentages were observed, indicating a lack of a concentration-dependent relationship in the blend’s oil protection.

### 3.6. Kinetic Evolution of the PBN Adduct Formation

**Figure 5:**
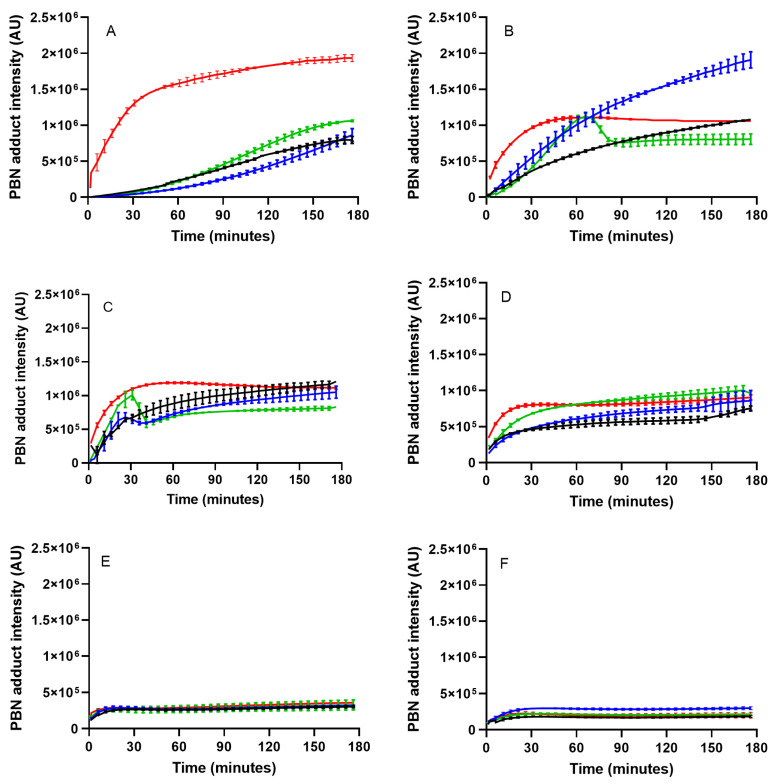
Evolution of PBN adduct intensity over time in in SFO (black line) and its blends at 5% (blue line), 10% (green line), and 15% (red line) with MSO extracted with 2-MeTHF during storage at 70 °C for 0 days (**A**), 2 days (**B**), 4 days (**C**), 7 days (**D**), 14 days (**E**), and 21 days (**F**).

Figure S2 and Figure 5 describe the evolution of the PBN adduct with time in oils (SFO and the blends) exposed to thermal treatment at 90 °C and previously subjected to accelerated aging.

In this paper, EPR spectroscopy was not used as a predictive analysis to evaluate the shelf life of a fresh oil but to study the oxidative status of the sample oils, even during storage at 70 °C, simulating longer storage time at room temperature. The evolution of the intensity of PBN adduct vs. time, together with parameters such as PV, AV, conjugated dienes, and trienes, provides valuable information about the oxidation degree of the oils. It is our opinion, in fact, that the parameters obtainable from spin trapping experiments with PBN cannot be used alone to establish the oxidative status of oils, but only in conjunction with other parameters related to primary and secondary oil oxidation products (PV, AV, K232, and K268).

In SFO, the amount of PBN adducts increased until the seventh day of accelerated aging up to reach, at the end of “storage”, the lowest value (Figure S2A).

The shape of the curves changed significantly during accelerated aging. At time 0, in oils not yet subjected to forced aging conditions, the PBN adduct increased slowly during the first 50 min of heating and then increased progressively with time. In contrast, in oil exposed to forced aging conditions for 2, 4, and 7 days, the PBN adduct increased immediately and reached values higher than that at time 0. As forced aging proceeded, the formation of the PBN adduct decreased significantly. The blends at 5 and 10% followed the same trend observed for SFO while, in the blend of 15% myrtle oil, the PBN adduct increased immediately at each forced aging time. Moreover, the highest increase was observed at the beginning of the experiment in oil not subjected to forced aging.

Figure 5 compares SFO and the blends at each forced aging time. At the beginning of the experiment (time 0), SFO and 5 and 10% blends had similar PBN adduct signal intensities while those measured for the 15% blend were significantly higher. In this case, the protective effect of the antioxidants present in the MSO is overcome by its high PV, which generates a greater amount of radical species trapped by PBN or by the pro-oxidant effect of the antioxidants when present in large quantities. The intensities of the 15% blend decreased progressively with aging. The intensities of the 5% and 10% blends increased after 2 days of forced aging at 70 °C then decreased to reach, at the 21st day of storage, quite similar values in all the samples.

These results show that PVs and AVs were not in accordance with the evolution of the PBN adduct with time.

As the curves representing the evolution of the intensity of the PBN adduct over time have different shapes, a direct quantitative comparison is not possible. It is useful to introduce another parameter representing the sum of the intensities of the PBN adducts during the first 180 min of heating at 90 °C. The AUC (area under the curve) parameter has been previously used in other contexts, for example, in beer samples subjected to thermal treatment at 60 °C in the presence of PBN [26].

**Figure 6:**
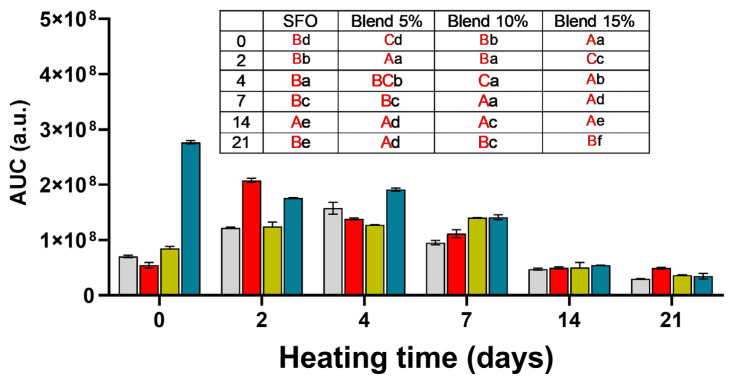
AUC values calculated for sunflower oil and its blends (5, 10, and 15%) with MSO extracted with 2-MeTHF during storage at 70 °C for 21 days. 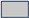 SFO; 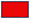 blend 5%; 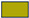
blend 10%; 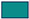 blend 15%. In the inset table: capital letters in red deal with the comparison between times inside the same blend; lowercase letters deal with the comparison between blends inside the same storage time. Mean differences were calculated according to Tukey’s test (*p* ≤ 0.05).

In SFO and in the blends at 5 and 10%, the accelerated aging conditions caused a rise in AUC values during the first 7 days of storage, followed by a decrease in the last 7 days (see Figure 6). The 5% blend showed a maximum AUC value before that of SFO and the 10% blend. On the contrary, the blend at 15% showed a progressive decrease during storage.

The blend at 15% showed, at the beginning of the experiment, the highest AUC value while, at the end of storage, its value was statistically indistinguishable from SFO. The high AUC value of the fresh 15% blend can be explained considering that the EPR experiments are performed at 90 °C; in these conditions, the oxygen availability is limited and, therefore, there is no significant formation of new hydroperoxides but mainly decomposition of those already present in the oil. The high PV of the MSO added explains this result.

As reported by Rahmani-Manglano et al. [27] and Falch et al. [28], not always a low concentration of radicals indicates a high oxidative stability. In bulk oil, samples that oxidize rapidly during forced aging were shown to have the lowest concentration of radicals [28].

In enriched oils, the system is even more complex. In this case, the adducts detected by EPR are the result of the equilibrium among the formation of radicals, their stability, their depletion by exogenous antioxidants, and the rate of formation and decomposition of the PBN adduct. Moreover, at the end of the storage period, when the antioxidants are presumably depleted, the PBN adducts may react with radical species, generating diamagnetic compounds and, therefore, silent EPR species. As shown in Figure S2, the intensity of the PBN adduct in oil stored for 21 days is always the lowest. In particular, in Figure S2D, the intensity of the PBN adduct decreases with storage time as oxidation increases.

The evolution of PVs, AVs, and AUC values during storage shows that the peroxides formed during the first stages of oxidation decompose into aldehydes and ketones. These compounds increase continuously in SFO while, in the blends, they start to rise from the 7th day of accelerated storage.

The trend of the oxidative parameters showed two well-defined phases. Up to the fourth day of accelerated storage, which, according to Chong et al. [29], corresponds approximately to 4 months of storage at room temperature, the blends seemed to be protected from oxidation, whereas from the seventh day of incubation at 70 °C, the AV and K268 values increased suddenly, indicating accelerated oxidation not inhibited by the antioxidants.

The AUC values reflect this trend. The low values observed starting from the 14th day indicate that the immediate precursors of radical species, i.e., hydroperoxides, are almost completely depleted, being converted into aldehydes and ketones. In addition, at 70 °C, when oxygen concentration is limited, the radical species arising from fatty acids preferentially do not form hydroperoxides but triglyceride polymers. The formation of polymeric species interrupts the radical chain reaction, preventing the formation of further hydroperoxides. At such long storage intervals, the different blends seem not to protect the oil against oxidation based on AUC values and the other parameters used to evaluate oil oxidation (PV, AV, K, and totox).

According to the parameters used to study the evolution of oil oxidation, blending with MSO protects SFO from oxidation regardless of the ratios tested. Even the 15% blend, although showing high levels of PVs, AVs, conjugated dienes and trienes during storage, seems to be able to protect SFO, as shown in Figure S1. The advantage of the 15% blend lies in its high concentration of phenolic compounds but the high concentration of peroxides limits its use. The 5% blend seems to give the best protection during forced aging, providing a good compromise between the antioxidants and peroxides added.

On the other side, if we consider the AUC values, the best blends seem to be 5 and 10%, even if the 5% blend at day 2 has an AUC value higher than SFO, while the 10% blend has AUC values always comparable to those of SFO.

A concentration dependence of the SFO oxidative stability protection with different MSO ratios (5%, 10%, 15%) seems not to be clearly present. This behavior may rely on the high amounts of peroxides present in MSO together with a high concentration of antioxidants. Moreover, the antioxidants may act as pro-oxidants when present in high concentrations, thus making a concentration-dependent effect more difficult to identify.

Further studies will be necessary to evaluate the potentialities of a refined MSO, considering that the drawback of the refining process is the loss of phenolic compounds and a possible reduction of oil antioxidant activity.

## 4. Conclusions

This article investigates the effect of blending SFO with an oil extracted sustainably from myrtle seeds, a by-product of the myrtle liqueur industry. In a previous paper, we showed that MSO extracted with 2-MeTHF, a low-toxicity and biodegradable solvent, has a high oxidative stability due to its high concentration of phenolic compounds [16].

Oil blending is an old approach employed to improve the quality and the oxidative stability of oils naturally prone to oxidation [3]. The novelty of this paper lies in the use of an oil extracted from a waste of the food industry. At present, MSO is not approved for human consumption, therefore, further tests are necessary to demonstrate its safety. The results demonstrated that the enrichment of SFO with 5 or 10% MSO effectively slowed down the oxidation in oils subjected to forced aging conditions at 70 °C for 21 days. The PVs of the 5% blends were always significantly lower than SFO. The addition of higher amounts of myrtle oil, as in 10%, slowed down the oxidation process until the seventh day of storage, whereas the addition of 15% myrtle oil did not determine any improvement in the oxidative stability of the blends. These results suggest for PVs a concentration effect that was not observed in the other parameters, like AV, totox, or K232 and K268. This lets us hypothesize that the antioxidants present in the blends, coming from either SFO or myrtle oil, influence the first stages of oxidation and, once they are depleted, are no longer able to protect oils from further oxidation.

In conclusion, the blending of SFO with MSO is a valuable method to extend its shelf life. Among the MSO concentrations tested, the blend at 5% showed the highest protective effect. These results may be useful to developing new protective strategies for edible oils subjected to storage or cooking, using unconventional oils extracted from seeds obtained from agricultural by-products, following a circular economy approach. However, MSO should probably be refined before being used in blending with edible oils to decrease their PVs, although, during the refining process, some antioxidants are lost. Further research is necessary to optimize the exploitation of MSO in the oxidative stabilization of edible oils.

## Data Availability

The original contributions presented in this study are included in the article/Supplementary Materials. Further inquiries can be directed to the corresponding authors.

## Supplementary Materials

The following supporting information can be downloaded at: https://www.mdpi.com/article/10.3390/antiox14030300/s1, Figure S1: Percentage of PV increase, relative to non-heated samples (time 0), measured for SFO and its blends (5, 10 and 15%) with MSO extracted with 2-MeTHF during storage at 70 °C for 21 days.; Figure S2: Evolution of PBN adduct intensity over time in SFO (A) and its blends at 5% (B), 10% (C) and 15% (D) with myrtle seed oil extracted with 2-MeTHF during storage at 70 °C for 0 (black line), 2 days (blue line), 4 days (red line), 7 days (green line), 14 days (grey line), 21 days (brown line).
