# Supplementary material for: Oxidative Stability of Sunflower Oil: Effect of Blending with an Oil Extracted from Myrtle Liqueur By-Product
Source: Antioxidants (Basel). 2025 Feb 28;14(3):300. doi: 10.3390/antiox14030300 (PMC11939490; doi:10.3390/antiox14030300)
Supplement: Supplementary file 1 [file antioxidants-14-00300-s001.zip › antioxidants-3479391-supplementary.pdf]

SUPPLEMENTARY MATERIAL

Oxidative stability of sunflower oil: effect of blending with an oil extracted from myrtle liqueur by-product

Daniele Sanna [1]* and Angela Fadda [2,]*

[1]Institute of Biomolecular Chemistry, National Research Council, Traversa La Crucca, 3, 07100 Sassari, Italy; daniele.sanna@cnr.it

[2]Institute of the Sciences of the Food Productions, National Research Council, Traversa La Crucca, 3, 07100 Sassari, Italy; angela.fadda@cnr.it

*Correspondence: angela.fadda@cnr.it; Tel.: +39-079-284-1714; daniele.sanna@cnr.it, Tel.: +39-079-284-1207

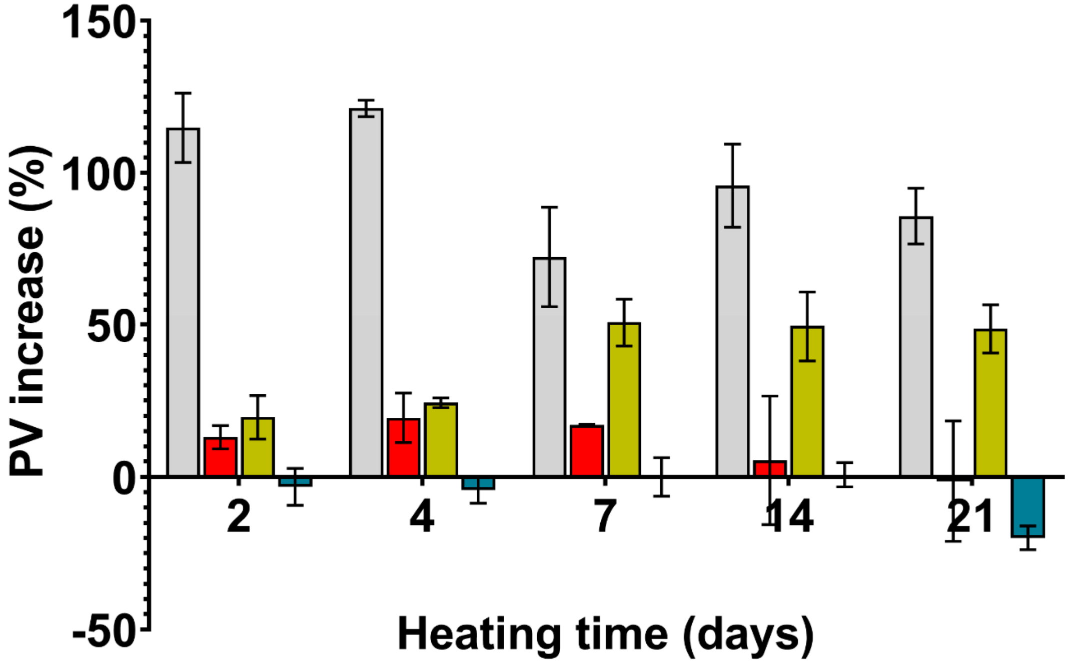

Figure S1. Percentage of PV increase, relative to non-heated samples (time 0), measured for SFO and its blends (5, 10 and 15%) with MSO extracted with 2-MeTHF during storage at 70 °C for 21 days.
SFO; blend 5%; blend 10%; blend 15%.

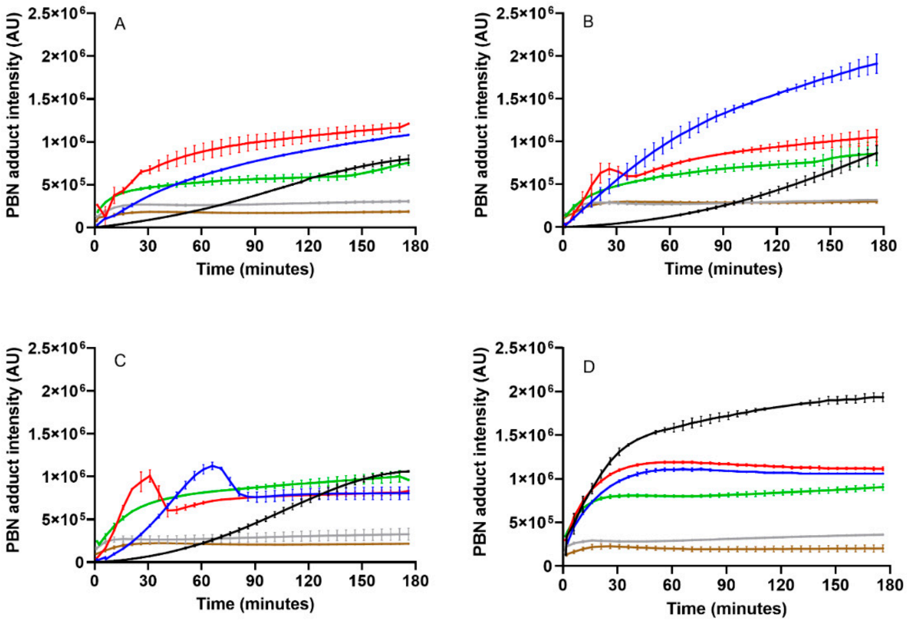

Figure S2. Evolution of PBN adduct intensity over time in SFO (A) and its blends at 5% (B), 10% (C) and 15% (D) with myrtle seed oil extracted with 2-MeTHF during storage at 70 °C for 0 (black line), 2 days (blue line), 4 days (red line), 7 days (green line), 14 days (grey line), 21 days (brown line).
